# Skin Temperature Change Following Infected and Non-infected Total Knee Arthroplasty: A Systematic Review

**DOI:** 10.7759/cureus.84549

**Published:** 2025-05-21

**Authors:** Fadel Jesry, Hemant Pandit, Dominic Clarke, Ramakrishnan Venkatesh

**Affiliations:** 1 Orthopaedics, Hull Royal Infirmary, Hull, GBR; 2 Orthopaedics, Leeds Institute of Rheumatic and Musculoskeletal Medicine, University of Leeds, Leeds, GBR; 3 Engineering, Invibio Ltd, University of Leeds, Leeds, GBR; 4 Orthopaedics, Leeds Teaching Hospitals Trust, Leeds, GBR

**Keywords:** prosthetic joint infection, skin temperature, systematic review, thermography, total knee arthroplasty

## Abstract

Total knee arthroplasty (TKA) is a common and growing surgical intervention for end-stage knee osteoarthritis. Postoperative changes in skin temperature (ΔST) over the operated knee are well recognised, yet their clinical relevance, particularly as a potential marker for periprosthetic joint infection (PJI), remains uncertain.

This systematic review aimed to define the pattern of skin temperature change following TKA and assess its potential utility in detecting PJI.

A comprehensive literature search was conducted using PubMed, EMBASE, Google Scholar, and the Cochrane Database up to April 2025, adhering to PRISMA (Preferred Reporting Items for Systematic Reviews and Meta-Analyses) guidelines. Eleven studies were included, covering a total of 1,212 patients. Inclusion criteria required objective ΔST measurements of the anterior knee in adults (≥18 years) with at least six months of follow-up. Studies on both uncomplicated TKA and TKA complicated by PJI were analysed narratively.

All non-infected cases demonstrated a marked rise in ΔST over the operated knee, peaking within the first week postoperatively with a weighted mean of 3.42°C, followed by a progressive decline to 0.9°C at six months and 0.48°C at one year. In contrast, PJI cases (n=25, conservatively managed) exhibited greater and more persistent ΔST elevations, particularly within the early postoperative period. At one week post-TKA, the infected group had a mean ΔST 0.78°C higher than the non-infected cohort. Patients undergoing revision for PJI (n=3) showed extreme elevations exceeding 4°C, sustained through the first three months postoperatively.

Skin temperature over the knee typically follows a predictable decline after TKA in uncomplicated cases. While elevated or prolonged ΔST may indicate infection, current evidence (limited by heterogeneous methods and small sample sizes) does not support ΔST as a standalone diagnostic marker for PJI. Further large-scale, standardised studies are required to explore its role in early infection detection.

## Introduction and background

Rationale

The volume of total knee arthroplasties (TKAs) performed is continuing to increase with population as life expectancy increases [1]. The rate of TKAs increased by 3.3 fold between 1991 and 2006 in the UK [2], with data from the National Joint Registry for England, Wales, Northern Ireland and the Isle of Man suggesting that by 2060, the demand for TKAs will increase by a further 40% from 2018 levels [3]. In the US, the growth rate is even higher, with projections of 3.5 million TKAs per annum within the next seven years, which would equate to a growth rate of 601% from 2005 levels [4].

Periprosthetic joint infections (PJIs) are a dangerous post-operative complication with a 1-2% incidence rate following primary arthroplasties [5]. Recent data (from 2020) shows that the annual costs associated with PJIs post-TKA within the US equates to $1.1 billion [6]. PJIs post-TKA are a devastating complication and early diagnosis is required to minimise morbidity and mortality [7]. Increased skin temperature (ST) around the knee has been associated with infection [8] and is commonly used as a surrogate measure by clinicians to assess a patient’s condition including the possibility of PJI [9]. At the same time, it is well known that the ST post uncomplicated TKA also shows variation, believed to relate to the healing process and in part due to the heat generated by the prosthesis which typically has metal components [10].

Objectives

The primary objective of this study is to understand the normal progression of ST change following TKA. The second objective is to evaluate the use of skin temperature as a surrogate marker for suspected infection post-TKA.

## Review

Methodology

Protocol

This systematic review was structured according to the Preferred Reporting Items for Systematic Reviews and Meta-Analyses (PRISMA) principles [11]. The title of the study was proposed in line with the Population, Intervention, Comparison and Outcome (PICO) guidelines [12]. Comparison would be made between the two groups (uncomplicated versus PJI complicated TKAs) across a variety of time frames. The outcome chosen was skin temperature change across multiple studies.

Eligibility Criteria

Inclusion criteria: Studies published from 2005 to 2025 reporting on measured and patient-reported ST change on the anterior aspect of the replaced knee in patients aged ≥ 18 with a history of primary TKA, confirmed cases of PJI and a minimum follow-up of six months. Studies must also be published in the English language.

Statistical Analyses

Given the lack of infected TKA data, no meta-analysis was done. Thus, narrative synthesis, tables and graphs were used to analyse the data.

Information Sources

The following information sources were used to review the literature: PubMed, EMBASE, Google Scholar and the Cochrane Database of Systematic Reviews.

Search Strategy

For both primary and secondary objectives the following inputs were used: (("total knee arthroplasty") OR ("total knee replacement")) AND (("skin temperature") OR ("skin temperature change")).

The initial database search yielded a total of 55 articles from four sources: PubMed (n=20), Cochrane Library (n=17), Google Scholar (n=10), and EMBASE (n=8). After removing 25 duplicate records, 30 articles remained for screening. An additional three records were identified through a citation search.

**Figure 1:**
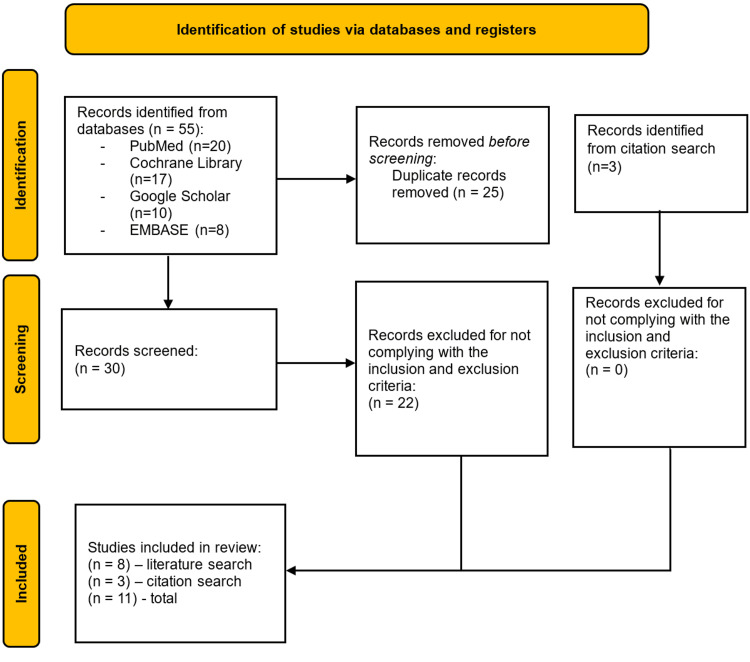
PRISMA flow chart PRISMA (Preferred Reporting Items for Systematic Reviews and Meta-Analyses) flow diagram representing the process of identifying, screening, and selecting studies for the systematic review [11]

Of the 30 screened records, 22 were excluded for not meeting the inclusion and exclusion criteria, leaving eight studies from the database search. All three citation search articles met the criteria and were included. In total, 11 studies were included in the final review. Figure 1 depicts the PRISMA flow diagram.

Selection of Sources of Evidence

Screening and eligibility were based on the aforementioned inclusion and exclusion criteria. This produced nine studies focused solely on changes in skin temperature in non-complicated TKAs [13-22] and two studies which included data on the changes in skin temperature in PJI-complicated TKAs [19,23].

Results

The majority of studies had a background of primary knee osteoarthritis (OA) and few had other clinical indications for TKA (e.g. traumatic knee osteoarthritis (KOA), osteonecrosis and rheumatoid arthritis). Follow-up intervals also varied between one day (1d) to one year (1y). Further details can be seen in Table 1.

**Table 1:** Characteristics of studies Included in the systematic review KOA, knee osteoarthritis; RA, rheumatoid arthritis; PJI, periprosthetic joint infection; TKA: total knee arthroplasty; M:F, male:female counts; Age, mean (years old); NR, not reported; d, day; w, week; m, month; y, year

Author (year)	Country (study period)	Sample size	Gender ratio M:F	Mean age (range)	Underlying condition(s)	Follow-ups
Mehra (2005) (non-infected) [13]	UK (NR)	19	M:F 7:13	Age 72 (44–85)	18 KOA, 1 RA	1w, 6w, 3m
Haidar (non-infected) (2006) [14]	UK (2001–2004)	32	M:F 10:22	Age 70 (59–85)	KOA	1w, 6w, 3m, 6 m, 1y, 2y
Martinez (non-infected) (2007) [15]	France (NR)	20	M:F 1:19	Age 69	KOA	1d, 4d, 1m, 4m
Martin (non-infected) (2008) [16]	France (NR)	18	M:F 4:14	Age 70	KOA	1d, 4d, 1w, 1m, 3 m
Honsawek (non-infected) (2011) [17]	Thailand (2007–2008)	49	M:F 9:40	Age 68 (50–78)	KOA	2w, 6w, 3m, 6 m
Romano (non-infected) (2011) [18]	Italy (2008)	40	M:F 28:12	Age 64 (53–78)	32 primary KOA; 4 traumatic KOA, 4 osteonecrosis	1d, 3d, 1w, 1m, 6w, 3m, 6 m, 1y
Mumingjiang (2014) (group 1 – normal) [19]	China (2012–2013)	21	M:F 5:16	Age 58 (25–72)	15 primary KOA; 3 post-traumatic KOA, 2 RA, 1 osteochondritis dissecans	1d, 7d, 1m, 3m, 6m
Mumingjiang (2014) (group 2 – PJI) [19]	China (2012–2013)	7	M:F NR	Age NR	NR	6 m
Mumingjiang (2014) (group 3 - PJI with TKA revision) [19]	China (2012–2013)	3	M:F NR	Age NR	NR	1d, 1w, 1m, 3m, 6m
van Hove (non-infected) (2015) [20]	Netherlands (2006–2007)	50	M:F 13:37	Age 68	44 primary KOA; 2 RA; 4 KOA + other	6w, 6m, 1y, 5y
Zeng (non-infected) (2016) [21]	China (2012–2014)	39	M:F 4:35	Age 67	KOA	1d, 3d, 5d, 1w, 2w, 1m, 3m, 6 m, 1y
Xu (2018) [22] (non-infected)	China (2016–2017)	60	M:F NR	Age 64	KOA	1d, 3d, 1w, 2w, 6w
Sharma (2024) (infected and non-infected) [23]	Canada (NR)	889 (Not infected: 864, infected: 25)	M:F 317:572	Not infected: Age 67 Infected: Age 70 Range: 31-90	NR	2w, 6w, 12w, 1y

The mean ST (°C) differences across various time points can be seen in Tables 2-8.

**Table 2:** Mean ΔST (°C) pre-TKA Δ, difference; ST, skin temperature; TKA, total knee arthroplasty; PCA: patient-controlled analgesia; CoCrMo, cobalt–chromium–molybdenum

Name	Mean ΔST pre-TKA (°C)	n size
Mehra (2005) [13]	0.1	19
Haidar (2006) [14]	0.1	32
Martinez (2007) [15]	0.5	20
Martin (PCA) (2008) [16]	0.7	18
Romano (2011) [18]	0.1	40
Mumingjiang (2014) [19]	1	21
van Hove (CoCrMo) (2015) [20]	0.1	50
Zeng (2016) [21]	0.5	39
Xu (Standard) (2018) [22]	0.6	60
Sharma non-infected (2024) [23]	0.3	864
Total mean normal TKA	0.32	1163
Revised TKA - Mumingjiang (2014) [19]	5.18	3
Sharma infected (2024) [23]	-0.15	25

**Table 3:** Mean ΔST (°C) one day post-TKA Δ, difference; ST, skin temperature; TKA, total knee arthroplasty; PCA, patient-controlled analgesia

Name	Mean ΔST day 1 (°C)	n size
Haidar (2006) [14]	2.9	32
Martin (PCA) (2008) [16]	3.3	18
Romano (2011) [18]	3.4	40
Mumingjiang (2014) [19]	3.9	21
Zeng (2016) [21]	1	39
Xu (Standard) (2018) [22]	2.5	60
Total mean normal TKA	2.82	210
Revised TKA - Mumingjiang G3 (2014) [19]	4.93	3

**Table 4:** Mean ΔST (°C) one to two weeks post-TKA Δ, difference; ST, skin temperature; TKA, total knee arthroplasty; PCA: patient-controlled analgesia

Name	Mean ΔST week 1-2 (°C)	n size
Mehra (2005) [13]	1.7	19
Martinez (2007) [15]	4.7	20
Martin (PCA) (2008) [16]	3	18
Romano (2011) [18]	3	40
Mumingjiang (2014) [19]	4.4	21
Zeng (2016) [21]	2.4	39
Xu (Standard) (2018) [22]	3.2	60
Sharma non-infected (2024) [23]	3.48	864
Total mean normal TKA	3.42	1081
Revised TKA - Mumingjiang G3 (2014) [19]	4.8	3
Sharma infected (2024) [23]	4.2	25

**Table 5:** Mean ΔST (°C) four to six weeks post-TKA Δ, difference; ST, skin temperature; TKA, total knee arthroplasty; PCA: patient-controlled analgesia; CoCrMo, cobalt–chromium–molybdenum

Name	Mean ΔST week 4-6 (°C)	n size
Mehra (2005) [13]	1.7	19
Haidar (2006) [14]	1.6	32
Martinez (2007) [15]	3.7	20
Martin (PCA) (2008) [16]	1	18
Honsawek (2010) [17]	3.5	49
Romano (2011) [18]	1.5	40
Mumingjiang (2014) [19]	3.7	21
van Hove (COCrMo) (2015) [20]	1.6	50
Zeng (2016) [21]	2.3	39
Xu (Standard) (2018) [22]	2.2	60
Sharma non-infected (2024) [23]	3	864
Total mean normal TKA	2.78	1212
Revised TKA - Mumingjiang G3 (2014) [19]	3.27	3
Sharma infected (2024) [23]	3.26	25

**Table 6:** Mean ΔST (°C) three to four months post-TKA Δ, difference; ST, skin temperature; TKA, total knee arthroplasty; PCA: patient-controlled analgesia

Name	Mean ΔST month 3-4 (°C)	n size
Mehra (2005) [13]	0	19
Haidar (2006) [14]	1.3	32
Martinez (2007) [15]	2.3	20
Martin (PCA) (2008) [16]	0.7	18
Honsawek (2010) [17]	2.7	49
Romano (2011) [18]	0.2	40
Mumingjiang (2014) [19]	2	21
Zeng (2016) [21]	1.8	39
Sharma non-infected (2024) [23]	2.18	864
Total mean normal TKA	2.01	1102
Revised TKA - Mumingjiang G3 (2014) [19]	1.08	3
Sharma infected (2024) [23]	2.35	25

**Table 7:** Mean ΔST (°C) six months post-TKA Δ, difference; ST, skin temperature; TKA, total knee arthroplasty; CoCrMo, cobalt–chromium–molybdenum; PJI, periprosthetic joint infection

Name	Mean ΔST month 6 (°C)	n size
Haidar (2006) [14]	0.3	32
Honsawek (2010) [17]	1	49
Romano (2011) [18]	0.2	40
Mumingjiang (2014) [19]	1.7	21
van Hove (CoCrMo) (2015) [20]	0.8	50
Zeng (2016) [21]	1.4	39
Total mean normal TKA	0.9	231
PJI post TKA - Mumingjiang G2 (2014) [19]	4.2	7
Revised TKA - Mumingjiang G3 (2014) [19]	0.79	3

**Table 8:** Mean ΔST (°C) one year post-TKA Δ, difference; ST, skin temperature; TKA, total knee arthroplasty; CoCrMo, cobalt–chromium–molybdenum

Name	Mean ΔST 1 year (°C)	n size
Haidar (2006) [14]	0.3	32
Romano (2011) [18]	0.1	40
van Hove (CoCrMo) (2015) [20]	1	50
Zeng (2016) [21]	0.9	39
Sharma non-infected (2024) [23]	0.46	864
Total mean normal TKA	0.48	903
Sharma infected (2024) [23]	0.67	25

**Figure 2:**
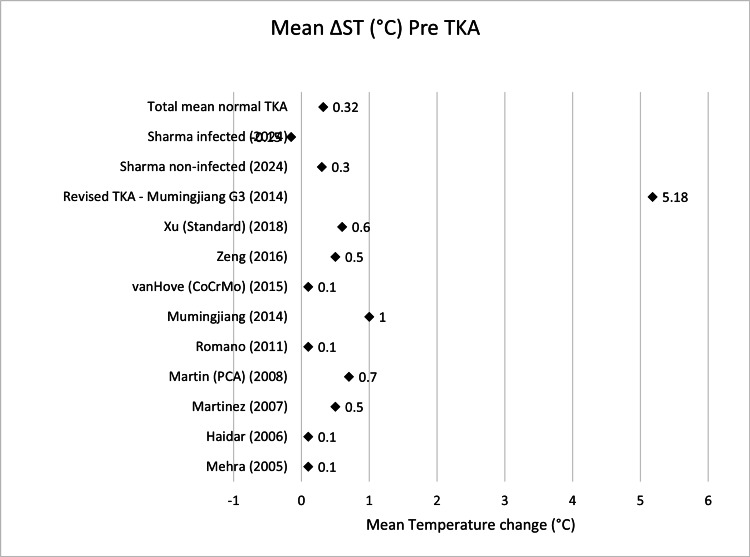
Scatter graph showing mean ΔST (°C) pre-TKA Δ, difference; ST, skin temperature; TKA, total knee arthroplasty; PCA: patient-controlled analgesia; CoCrMo, cobalt–chromium–molybdenum Mehra (2005) [13], Haidar (2006) [14], Martinez (2007) [15], Martin (PCA) (2008) [16], Romano (2011) [18], Mumingjiang (2014) [19], van Hove (CoCrMo) (2015) [20], Zeng (2016) [21], Xu (Standard) (2018) [22], Revised TKA - Mumingjiang G3 (2014) [19], Sharma non-infected (2024) [23], Sharma infected (2024) [23]

**Figure 3:**
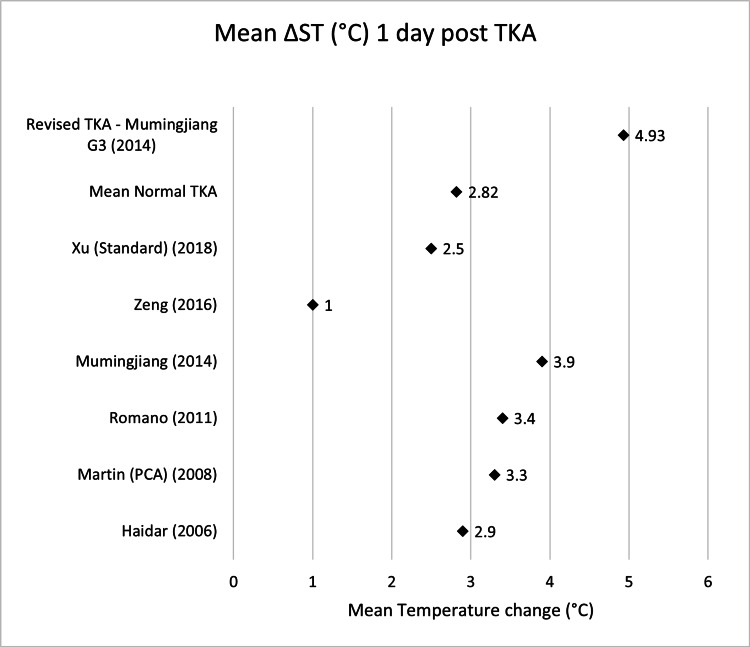
Scatter graph showing mean ΔST (°C) one day post-TKA Δ, difference; ST, skin temperature; TKA, total knee arthroplasty; PCA: patient-controlled analgesia Haidar (2006) [14], Martin (PCA) (2008) [16], Romano (2011) [18], Mumingjiang (2014) [19], Zeng (2016) [21], Xu (Standard) (2018) [22], Revised TKA - Mumingjiang G3 (2014) [19]

**Figure 4:**
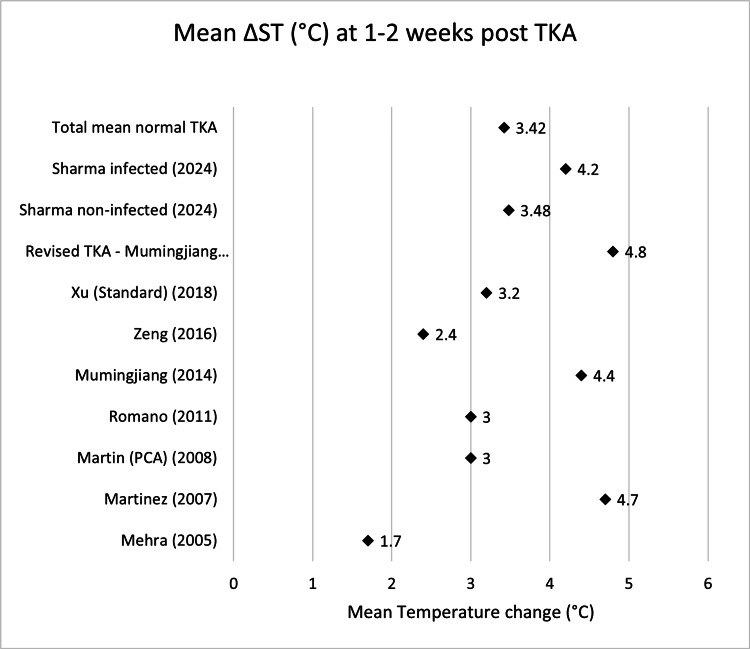
Scatter graph showing mean ΔST (°C) one to two weeks post-TKA Δ, difference; ST, skin temperature; TKA, total knee arthroplasty; PCA: patient-controlled analgesia Mehra (2005) [13], Martinez (2007) [15], Martin (PCA) (2008) [16], Romano (2011) [18], Mumingjiang (2014) [19], Zeng (2016) [21], Xu (Standard) (2018) [22], Revised TKA - Mumingjiang G3 (2014) [19], Sharma non-infected (2024) [23], Sharma infected (2024) [23]

**Figure 5:**
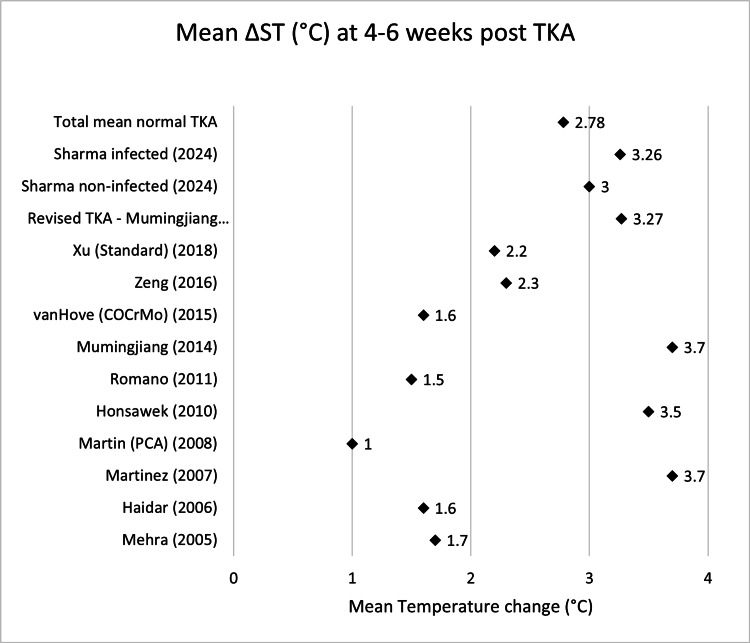
Scatter graph showing mean ΔST (°C) four to six weeks post-TKA Δ, difference; ST, skin temperature; TKA, total knee arthroplasty; PCA: patient-controlled analgesia; CoCrMo, cobalt–chromium–molybdenum Mehra (2005) [13], Haidar (2006) [14], Martinez (2007) [15], Martin (PCA) (2008) [16], Honsawek (2010) [17], Romano (2011) [18], Mumingjiang (2014) [19], van Hove (CoCrMo) (2015) [20], Zeng (2016) [21], Xu (Standard) (2018) [22], Revised TKA - Mumingjiang G3 (2014) [19], Sharma non-infected (2024) [23], Sharma infected (2024) [23]

**Figure 6:**
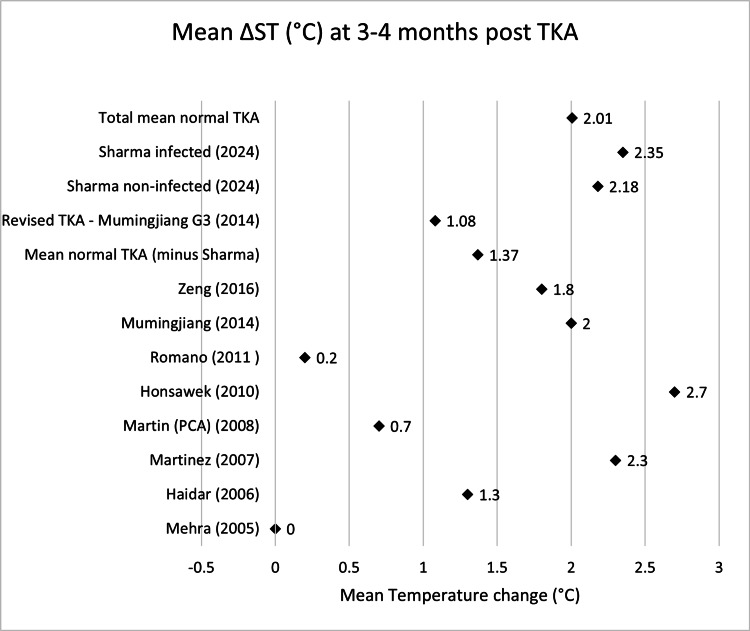
Scatter graph showing mean ΔST (°C) three to four months post-TKA Δ, difference; ST, skin temperature; TKA, total knee arthroplasty; PCA: patient-controlled analgesia Mehra (2005) [13], Haidar (2006) [14], Martinez (2007) [15], Martin (PCA) (2008) [16], Honsawek (2010) [17], Romano (2011) [18], Mumingjiang (2014) [19], Zeng (2016) [21], Revised TKA - Mumingjiang G3 (2014) [19], Sharma non-infected (2024) [23], Sharma infected (2024) [23]

**Figure 7:**
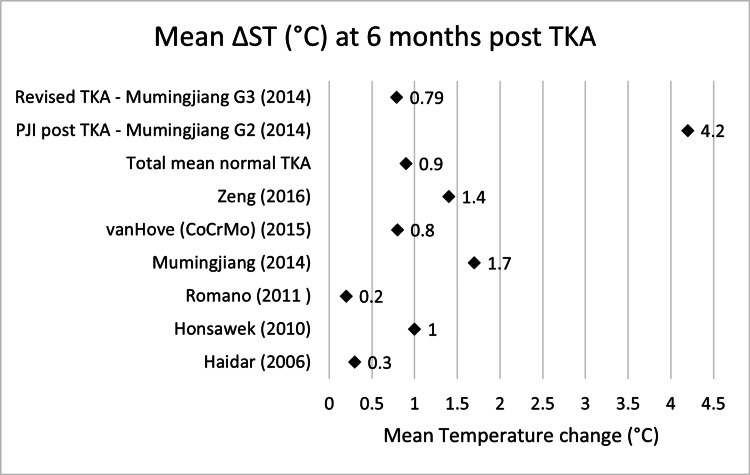
Scatter graph showing mean ΔST (°C) six months post-TKA Δ, difference; ST, skin temperature; TKA, total knee arthroplasty; CoCrMo, cobalt–chromium–molybdenum; PJI, periprosthetic joint infection Haidar (2006) [14], Honsawek (2010) [17], Romano (2011) [18], Mumingjiang (2014) [19], van Hove (CoCrMo) (2015) [20], Zeng (2016) [21], PJI post TKA - Mumingjiang G2 (2014) [19], Revised TKA - Mumingjiang G3 (2014) [19]

**Figure 8:**
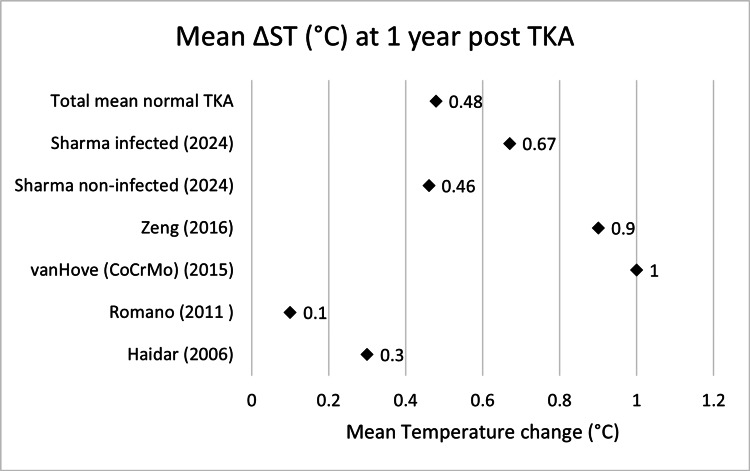
Scatter graph showing mean ΔST (°C) one year post-TKA Δ, difference; ST, skin temperature; TKA, total knee arthroplasty; CoCrMo, cobalt–chromium–molybdenum Haidar (2006) [14], Romano (2011) [18], van Hove (CoCrMo) (2015) [20], Zeng (2016) [21], Sharma non-infected (2024) [23], Sharma infected (2024) [23]

Scatter graphs showing mean ΔST (°C) across various time points can be seen in Figures 2-8. 

**Figure 9:**
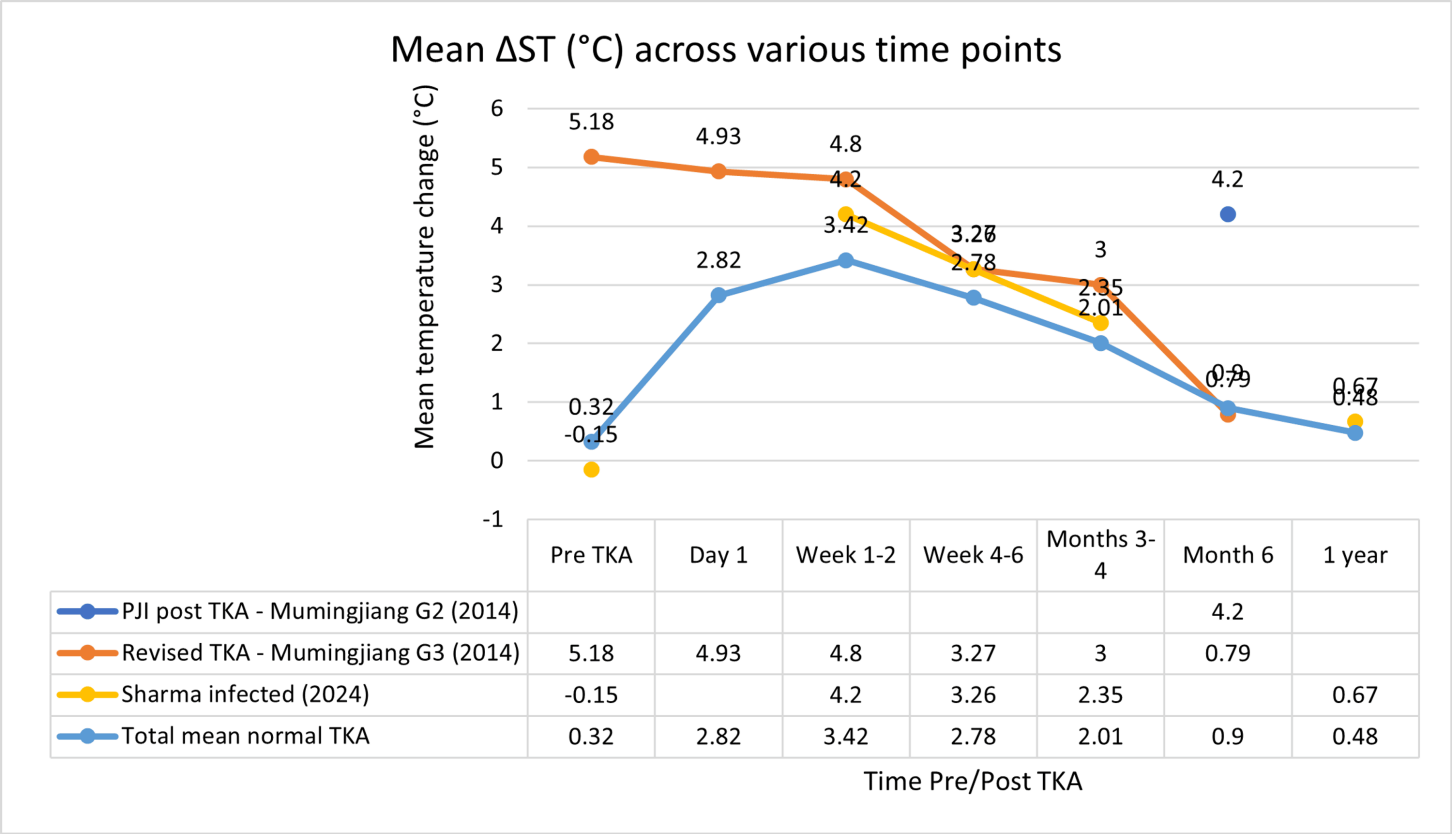
Scatter graph showing weighted means of ΔST (°C) across various time points with data table Δ, difference; ST, skin temperature; PJI, periprosthetic joint infection PJI post TKA - Mumingjiang G2 (2014) [19], Revised TKA - Mumingjiang G3 (2014) [19], Sharma infected (2024) [23]

The scatter graph showing weighted means of ΔST (°C) across various time points with a data table can be seen in Figure 9.

Eleven studies, comprising a total of 1,212 patients, were included in this systematic review. All studies investigated postoperative skin temperature changes in the knee following TKA, comparing the operated knee to the contralateral, non-operated side. Sample sizes varied from as few as three patients in revision cases to 889 in the largest cohort. All temperature measurements were reported in degrees Celsius.

In non-infected patients, all studies reported a postoperative elevation in skin temperature over the operated knee. This elevation was detectable as early as the first postoperative day and was consistent across the various study populations. Martin et al. (2008) (n=18) reported a mean increase of 3.3°C on postoperative day 1, which decreased to 1°C at one month and further to 0.7°C at four months [16]. Romano et al. (2011) (n=40) documented a temperature difference of 3°C at one week, falling to 1.5°C at one month, 0.2°C at three months, and 0.2°C at six months [18].

Honsawek et al. (2011) (n=49) measured skin temperature differences at six weeks, three months and six months, reporting values of 3.5°C, 2.7°C, and 1°C respectively [17]. Zeng et al. (2016) (n=39) reported a temperature difference of 1°C on postoperative day 1, 2.4°C by week one, 2.3°C at week six, 1.8°C by three months, 1.4°C at six months and 0.9°C at one year [21]. Xu et al. (2018) (n=60) found a peak difference of 2.4°C on day 1, declining to 1.5°C by day 3, 1.1°C at one week, 0.8°C at two weeks, and 0.5°C at six weeks [22]. Further details can be found in Tables 2-8 and Figures 2-9.

In contrast, studies reporting on infected cases demonstrated both higher temperature elevations and prolonged durations of abnormal thermal patterns. Mumingjiang et al. (2014) observed a mean difference of 4.2°C at six months in infected patients without revision TKA (n=7), and 0.79°C in infected patients having undergone previous TKA (n=3), compared to 1.7°C in the non-infected group (n=21) at the same time point [19]. In patients undergoing revision for prosthetic joint infection (n=3), elevated temperatures persisted throughout the six-month period including before the revision procedure, from 5.18°C pre-TKA, 4.93°C at one day, 4.8°C at one week, 3.27°C at six weeks, 3°C at three months and decreased down to 0.79°C at six months. At six months the revised TKA group showed a lower mean temperature change (0.79°C, n=3) than both the Mumingjiang control group (1.7°C, n=21) and the total weight mean of non-infected knees (0.9°C, n=231).

Sharma et al. (2024) conducted the largest cohort study (n=889) and stratified data between infected (n=25) and non-infected (n=864) patients [23]. Pre-TKA the infected group had a -0.15°C difference compared to the 0.3°C seen in the non-infected group. At two weeks, the infected group demonstrated a mean temperature difference of 4.2°C compared to 3.48°C in the non-infected group. The differences then are far smaller at six weeks (3.26°C infected vs 3°C non-infected), three months (2.35°C infected vs 2.18°C non-infected) and one year (0.67°C infected vs 0.46°C) respectively.

Collectively, these findings indicate that in non-infected TKA patients, skin temperature typically peaks within the first postoperative week with the weighted mean being 3.42°C. This elevation declines progressively, often approaching baseline by the sixth month (0.9°C) to one year (0.48°C). However, in infected cases (conservatively managed, n=25) when compared to the weighted mean (n>1000), they are initially 0.47°C cooler pre-TKA then 0.78°C warmer at one-week post-TKA, 0.48°C warmer at six weeks, 0.34°C warmer at three months and 0.19°C warmer at one year. This is quite different to the prolonged elevation seen when comparing infected TKAs undergoing revision (n=3) with their non-infected counterparts from pre-TKA (4.86°C) to three months post-TKA (0.99°C) with six months post-TKA showing they are 0.11°C cooler.

Discussion

Use of ΔST to Diagnose PJI Post-TKA

Early detection and treatment of postoperative PJIs are extremely important as they require complicated therapies that typically include prolonged courses of antibiotics and the potential for revision surgeries [5]. There are three main time scales for PJIs post-operatively; early (0-3 months), delayed (3-24 months) and late (>2 years) [9]. Diagnosis of PJI cannot rely solely on the clinical assessment; recent collaborative criteria written by the European Bone and Joint Infection Society (EBJIS) do not include skin temperature as a criterion but rather only include the following clinical findings: recent fevers, purulent discharge, and sinus tracts with joint communication [24]. The remainder of the criteria largely rely on either imaging modalities (e.g. radiological signs of loosening) or laboratory sample testing (e.g. serum CRP, serum leucocyte count and aspiration culture with gram stain) [24]. To see the full criteria refer to Table 9 below.

**Table 9:** EBJIS PJI criteria EBJIS, European Bone and Joint Infection Society; PJI: prosthetic joint infection; PMN: polymorph neutrophils; CFU/ml, colony-forming units per millilitre; HPF, high power field Adapted from [24]. Open access article, distributed under the terms of the Creative Commons CC-BY license, which permits unrestricted use, distribution, and reproduction in any medium, provided the original work is properly cited.

	Infection unlikely (all findings negative)	Infection likely (two positive findings)	Infection confirmed (any positive finding)
Clinical and blood workup			
Clinical features	Clear alternative reason for implant dysfunction (e.g. fracture, implant breakage, malposition, tumor)	1) Radiological signs of loosening within the first five years after implantation 2) Previous wound healing problems 3) History of recent fever or bacteraemia 4) Purulence around the prosthesis	Sinus tract with evidence of communication to the joint or visualization of the prosthesis
C-reactive protein		> 10 mg/L (1 mg/dL)	
Synovial fluid cytological analysis			
Leukocyte count (cells/µL)	≤ 1,500	> 1,500	> 3,000
PMN (%)	≤ 65%	> 65%	> 80%
Synovial fluid biomarkers			
Alpha-defensin			Positive immunoassay or lateral-flow assay
Microbiology			
Aspiration fluid		Positive culture	
Intraoperative (fluid and tissue)	All cultures negative	Single positive culture	≥ Two positive samples with the same microorganism
Sonication (CFU/ml)	No growth	> 1 CFU/mL of any organism	> 50 CFU/mL of any organism
Histology			
High-power field (400x magnification)	Negative	Presence of ≥ five neutrophils in a single HPF	Presence of ≥ five neutrophils in ≥ five HPF
			Presence of visible microorganisms
Others			
Nuclear imaging	Negative three-phase isotope bone scan	Positive WBC scintigraphy	

Limitations of Review

There are many limitations when it comes to the data set of the complicated TKAs (both with PJI only (Group 2) and PJI resulting in revision (Group 3)) by Mumingjiang et al. (2014) [19]. The first issue to address is the sample size; with only seven in the prior and three in the latter. This may render the study subject to bias and lack of reproducibility. The second limitation is the lack of consistency with the timing of data collections, i.e., Group 2 was only compared at six months whereas Group 3 was compared at one day, one week, one month, three months and six months. This led to a skewed data set and loss of invaluable information as skin temperature is of more importance in early PJIs (within the first three months post-surgery) [9]. The third limitation is that neither Group 2 nor Group 3 included patient demographics (e.g., age and gender ratio) or underlying conditions (e.g., OA or RA).

However, the dataset from Sharma et al. (2024) included a significantly larger sample of conservatively managed infected TKAs (25 cases), but it did not include information on revised infected TKAs [23]. Additionally, the dataset lacked details on patient demographics and the indications for surgery.

This leads to the data provided having reduced value because the differences in temperature could be easily explained by a patient’s medical background (e.g. poorly controlled type 2 diabetes mellitus or immunosuppression secondary to advanced systemic lupus).

In addition, the data between Sharma et al. (2024) [23] and Mumingjiang et al. (2014) [19] were not very comparable given that Sharma exclusively included conservatively managed PJI’s post-TKA where Mumingjiang et al. (2014) [19] only included this at one time point (six months). Given Sharma et al. (2024) [23] did not collect data at this time point, it gives limited value in comparing the studies.

Patients across studies were not fully comparable due to differences in measurement techniques, control of body mass index (BMI), room temperature, and other environmental factors.

Sharma et al. (2024) used infrared thermometry at five fixed time points in 1,094 patients but did not report standardization of room temperature, patient positioning, or BMI control [23]. The anatomical sites of measurement were not specified, limiting reproducibility.

Haidar et al. (2006) conducted daily measurements using calibrated thermometers at a consistent anatomical site (2 cm superomedial to the patella) and standardized the time of day (midday), enhancing intra-patient reliability [14]. However, they did not control for BMI or ambient room temperature.

Honsawek et al. (2011) measured skin temperature using surface thermometers across four patellar landmarks, averaging the values [17]. They ensured consistency in measurement timing and thermometer use per patient but did not report ambient temperature or BMI data.

Zeng et al. (2016) used high-precision infrared thermometers (±0.4°C error margin) under controlled conditions, including seated positioning and ambient temperature (0-50°C, 10-95% humidity) [21]. They reported BMI and ASA scores, allowing some adjustment for patient factors, though environmental variability was still possible.

Mumingjiang et al. (2014) also demonstrated stronger methodological control than several other studies [19]. They used infrared thermography at predefined time points (Day 1, Day 7, and Months 1, 3, and 6), and compared 21 uncomplicated TKA patients to infected and revision groups. Measurements were taken under consistent conditions, but precise details on room temperature control were not specified. Importantly, BMI and comorbidities were considered as part of the patient selection criteria, and exclusion criteria included systemic inflammatory conditions or recent infections. This adds a level of comparability often absent in other studies, though variations in environmental standardization may still introduce bias.

Overall, methodological heterogeneity (particularly in temperature control, BMI consideration, and anatomical standardization) limits comparability across studies.

## Conclusions

This systematic review demonstrates a consistent increase in skin temperature (ΔST) over the operated knee following total knee arthroplasty (TKA), peaking within the first postoperative week and gradually returning toward baseline by six to 12 months. In uncomplicated cases, the thermal rise averages 3.42°C and declines predictably, reflecting a physiological trajectory of inflammation and healing. In contrast, cases involving periprosthetic joint infection (PJI) show greater and more sustained thermal elevations. Infected knees, particularly those requiring revision surgery, exhibit ΔST differences exceeding 4°C in the early phase, with persistently elevated temperatures compared to non-infected cases.

Although these trends suggest potential diagnostic value, the clinical utility of ΔST remains limited by inconsistent measurement protocols, varied assessment timelines, and inadequate reporting of patient demographics and clinical subgroups. Skin temperature monitoring may still serve as a useful adjunct in the early detection of PJI, particularly when interpreted alongside other clinical indicators. Future research should focus on large, well-characterised cohorts to establish standardised thresholds and integrate ΔST into multimodal diagnostic algorithms.
